# Single-Cell RNA Sequencing Analysis of Steroidogenesis and Spermatogenesis Impairment in the Testis of db/db Mice

**DOI:** 10.1155/2024/8797972

**Published:** 2024-05-23

**Authors:** Yun Hu, Ting-Ting Cai, Reng-Na Yan, Bing-Li Liu, Bo Ding, Jian-Hua Ma

**Affiliations:** ^1^Department of Endocrinology, The Affiliated Wuxi People's Hospital of Nanjing Medical University, Wuxi People's Hospital, Wuxi Medical Center, Nanjing Medical University, Wuxi, China; ^2^Department of Endocrinology, Nanjing First Hospital, Nanjing Medical University, Nanjing, Jiangsu, China

## Abstract

**Objective:**

The mechanism of steroidogenesis and spermatogenesis impairment in men with type 2 diabetes remains unclear. We aimed to explore the local changes of steroidogenesis and spermatogenesis in the testis of db/db mice. *Research Design and Methods*. We performed single-cell RNA sequencing analysis in the testis of db/db and C57BL/6J mice. The differentially expressed genes were then confirmed by real-time PCR. The histopathological characteristics of testis in db/db mice and C57BL/6J control were also performed.

**Results:**

The 20-week-old db/db mice had significantly higher blood glucose and body weight (both *p* < 0.001). The serum testosterone levels (4.4 ± 0.8 vs. 9.8 ± 0.7 ng/ml, *p*=0.001) and weight of the testis (0.16 ± 0.01 vs. 0.24 ± 0.01 g, *p* < 0.001) were significantly lower in db/db mice than that in C57BL/6J controls. db/db mice had a lower cross-sectional area of seminiferous tubules and thickness of the cell layer (both *p* < 0.05). The numbers of Sertoli cells and Leydig cells decreased in db/db mice (both *p* < 0.01). Single-cell RNA sequencing analysis showed that compared with the control group, the percentage of spermatogonia was significantly higher in the db/db mouse (*p* < 0.001), while the proportions of spermatocytes, round and elongating spermatids, and sperms were all lower in the db/db mouse (*p* all < 0.001). The most differentially expressed genes were found in round spermatids (*n* = 86), which were not found in spermatogonia, spermatocyte, and sperm. Igfbp5 was the most significantly decreased gene in Leydig cells of the db/db mouse, while the expression of Cd74, H2-Aa, and H2-Eb1 was elevated. Ccl7 and Ptgds were the most significantly increased and decreased genes in Sertoli cells of the db/db mouse.

**Conclusions:**

The present study indicates spermiogenesis and steroidogenesis defects in db/db mice. The mechanism of steroidogenesis impairment in the testis of db/db mice deserves further investigation.

## 1. Introduction

The incidence of type 2 diabetes (T2D) in men of reproductive age has been increasing recently, which is attributed to unhealthy diet and sedentary behavior. Poor glycemic control in men with T2D can lead to testosterone reduction and male sexual function impairment [1, 2]. The proportion of testosterone deficiency in men with T2D was significantly higher than that in normal persons [3]. Previous studies in both humans and animals also confirmed the deleterious effect of diabetes on spermatogenesis [4, 5]. Therefore, the disturbance in the male reproductive system is now considered an important complication of diabetes [4]. On the other hand, testosterone plays an important role in the regulation of carbohydrate, fat, and protein metabolism. Recently, several clinical studies have demonstrated that testosterone replacement therapy has positive effects on the prevention and reversal of T2D [6–8]. Addressing the problem of testosterone deficiency in males with T2D will contribute to their glycemic control.

The mechanism of steroidogenesis and spermatogenesis impairment in men with T2D remains unclear. The studies were mainly focusing on the disturbance of the hypothalamic-pituitary-gonad axis caused by obesity, insulin resistance, and inflammation [9, 10]. Others demonstrated that inflammation and oxidative stress may directly damage the plasma membrane and DNA of sperm, thereby inhibiting the fertility of males with diabetes [11–13], which were mainly carried out in streptozotocin (STZ) induced animal models. However, STZ may damage testicular cells directly. Leptin receptor-deficient db/db mice are commonly used mice models mimicking the conditions of type 2 diabetes development. In addition to abnormal blood glucose, db/db mice also have obesity, insulin resistance, and chronic inflammation [14], which are also the causes of testosterone deficiency in men with type 2 diabetes [15]. Moreover, a previous study has reported impaired steroidogenesis in the testis of leptin-deficient mice (ob/ob −/−) [16], and leptin receptor expression is characteristic of mature Leydig cells [17]. However, the change in steroidogenesis and spermatogenesis in db/db mice is rarely reported [18, 19].

Here, we explored the local changes of steroidogenesis and spermatogenesis in the testis of the db/db mouse using single-cell RNA sequencing analysis, which has emerged as a useful tool to identify transcriptional signatures of major germ and somatic cell types of the testis [20].

## 2. Research Design and Methods

### 2.1. Animals

All animal procedures were authorized and specifically approved by the Institutional Ethical Committee of Nanjing First Hospital. Mice were maintained in a temperature-, humidity-, and light-controlled environment (12 h light/dark cycle). They had free access to food (chow diet) and water.

All mice were purchased from the Comparative Medicine Centre of Yangzhou University (China). Six db/db mice with fasting blood glucose higher than 16.7 mmol/L and six C57BL/6J mice with normal blood glucose were used in this study. Blood glucose was measured by using a J & J OneTouch glucometer. At 20 weeks old, all mice were weighed, and blood samples were collected from the atria after pentobarbital (1.5%) anesthesia. Testosterone levels were tested using the ELISA assay. The testis from one db/db mouse with low testosterone level and one C57BL/6J mouse with normal testosterone were used for single-cell RNA sequencing, while the others were collected for PCR analysis and histopathological analysis.

### 2.2. Cell Dissociation

The testis were dissected, washed with cold phosphate-buffered saline (PBS; Thermo Fisher Scientific, Waltham, MA, USA), cut into small pieces (<1 mm^3^), and transferred into 5 mL Dulbecco's modified Eagle's medium (DMEM; Thermo Fisher Scientific) containing 0.2% Collagenase Type I (Sigma-Aldrich) and 7.5 mg/mL DNase I (Sigma-Aldrich) [21]. Samples were incubated at 37°C in a water bath for 20 min, shaking every 5–10 min. At the end of the incubation time, a PBS-based wash buffer with 0.5% BSA (Sigma-Aldrich) was added to the digested tissue suspension and filtered through a 40 mm cell strainer. Samples were centrifuged at 300 g for 7 min. The washing step was repeated once more. Next, the cells were counted and assessed for viability using Trypan blue staining on a haemocytometer. When the viability of cells was higher than 80 percent, the experiment of library construction was performed.

### 2.3. Single Cell RNA-Seq Library Construction and Sequencing

The single-cell library was constructed using the ChromiumTM Controller and ChromiumTM Single Cell 3′ Reagent Version 2 Kit (10x Genomics, Pleasanton, CA). In brief, single cells, reagents, and gel beads containing barcoded oligonucleotides were encapsulated into nanoliter-sized GEMs (gel beads in emulsion) using the GemCode Technology. Lysis and barcoded reverse transcription of polyadenylated mRNA from single cells were performed inside each GEM. Post-RT-GEMs were cleaned up and cDNA were amplified. cDNA was fragmented and the fragments end were repaired, as well as A-tailing was added to the 3′ end. The adaptors were ligated to fragments which were double-sided SPRI selected. Another double-sided SPRI selecting was carried out after sample index PCR. The final library was quality and quantitated by two methods as follows: check the distribution of the fragments size using the Agilent 2100 bioanalyzer and quantify the library using real-time quantitative PCR (QPCR) (TaqMan Probe). The final products were sequenced using the DNBSEQ platform (BGI, Shenzhen, China).

### 2.4. Single Cell RNA-Seq Data Processing and Analysis

We used Cell Ranger (version 5.0.1) to generate a raw gene expression matrix for each scRNA-seq sample. Quality filtering was performed with Seurat (3.0.2) to remove cells with <200 expressed genes, or >90% of the maximum gene number, or >15% unique molecular identifiers (UMIs) derived from the mitochondrial genome. The cell cycle was also adjusted.

Then, we performed clustering of cells according to a previous study [22]. In brief, the cells were distinguished through markers as follows: somatic cells (Vim), spermatogonia (Sohlh1 and Utf1), spermatocytes (Piwil1 and Aurka), round spermatids: (Acrv1 and Nme5), elongating spermatids (Akap14 and Tnp2), sperm (Crisp2, Oaz3, and Prm2), Sertoli cells (Amh and Clu), immature Leydig cells (Inhba), Leydig cells (Cyp11a, Hsd3b1, and Insl3), myoid cells (Acta2 and Tagln), endothelial cells (Vwf), macrophage (Cd74 and Itgam), and T cells (Ccl5 and Cd247). The clustering results were shown using uniform manifold approximation and projection (UMAP) [23] plots.

The differences of gene expression, KEGG pathway, gene ontology (GO) enrichment, and cell communication between db/db mouse and C57BL/6J control were then analyzed in each cluster. According to the KEGG pathway and GO classification, we used the phyper function in R software to perform the enrichment analysis, calculate the *p* value, and the Q value was obtained by correction of the *p* value. Generally, the function of *Q* value <0.05 is regarded as a significant enrichment.

Cell-cell communication based on single-cell RNA-Seq data was analyzed using cellphoneDB [24]. Enriched receptor-ligand interactions between two cell types were derived based on the expression of a receptor by one cell type and the expression of the corresponding ligand by another cell type. The most relevant cell type-specific interactions between ligands and receptors were identified. We first randomly permuted the cluster labels of all cells 1000 times to determine the mean of the average receptor and ligand expression levels of the interacting clusters. This generated a null distribution for each receptor-ligand pair. By calculating the proportion of the means that were higher than the actual mean, a *p* value for the likelihood of the cell type specificity of the corresponding receptor-ligand complex was obtained.

### 2.5. RNA Extraction and Quantitative Real-Time PCR

Total RNA was extracted from testis using TRIzol reagent (Thermo Fisher Scientific, Waltham, MA, USA) and reverse transcribed into complementary DNA (cDNA) with the HiScript III RT SuperMix for qPCR (Vazyme Biotech Co., Ltd. Nanjing, CN), according to the protocol per manufacturer's instruction. Quantitative real-time PCR (qPCR) was performed with ChamQ Universal SYBR qPCR Master Mix (Vazyme Biotech Co., Ltd.) on an Applied Biosystems 7500 Real-Time PCR system (Applied Biosystems, Foster City, CA, USA). The samples were diluted to the same final cDNA concentration. The total reaction volume was 20 *μ*L, and the PCR was performed by using an initial incubation for 30 s at 95°C followed by 40 thermal cycles of 5 seconds at 95°C and 34 seconds at the annealing temperature of 60°C. GAPDH was used as an endogenous control for the genes. For each gene, the melting curve was analyzed to confirm the amplification of a single PCR product. The primer sequences are listed in Table 1, and the amplification efficiency was >90% for all genes. Relative expression levels were calculated using the cycle threshold (2^−ΔΔCt^) method. All results were confirmed by performing at least 2 additional independent runs.

### 2.6. Histopathological Analysis

We collected testis from four mice in each group for histopathological analysis, and at least three fields of view were analyzed in each testis. Testis tissues were fixed in 4% paraformaldehyde, dehydrated, paraffin embedded, sectioned at 4 *μ*m thickness, and subsequently stained with hematoxylin and eosin (H&E). We used the CaseViewer2.4 scanning software to select the target area of the tissue for 400x imaging and analyzed the histopathological characteristics using Image-Pro Plus 6.0 software. The area and diameter of the tubule were measured separately in each picture. The thickness of the cell layer was measured at 3 locations in each picture. Total cell number, Sertoli cells, and Leydig cells number per square millimeter were counted.

### 2.7. Statistical Analysis

Data were analyzed using SPSS statistical software (IBM Co., NY, USA). Statistical significance was determined by a Student's *t*-test. Data are shown as the mean ± SEM. Statistical significance was based on *p* < 0.05.

## 3. Results

### 3.1. The Characteristics of db/db Mice

The 20-week-old db/db mice had significantly higher blood glucose than controls (31.2 ± 1.0 vs. 10.6 ± 0.3 mmol/L, *p* < 0.001), as well as body weight (56.7 ± 0.7 vs. 27.8 ± 0.4 g, *p* < 0.001). The serum testosterone levels were significantly lower in db/db mice than that in C57BL/6J controls (4.4 ± 0.8 vs. 9.8 ± 0.7 ng/ml, *p*=0.001). The weights of testis in db/db mice were also significantly decreased (0.16 ± 0.01 vs. 0.24 ± 0.01 g, *p* < 0.001).

### 3.2. The Histopathological Characteristics of Testis in db/db Mice

**Figure 1:**
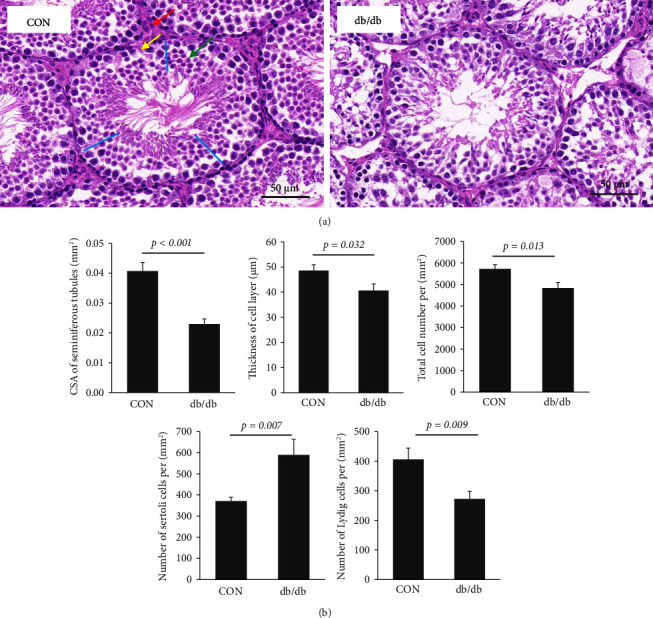
The histopathological characteristics of testis in db/db mice. (a) Testis tissues from four C57BL/6J mice (CON) and four db/db mice (db/db) were stained with hematoxylin and eosin. The red arrow indicates a Leydig cell, and the yellow arrow indicates a Sertoli cell. The green arrow shows a spermatid, which together with spermatogonia and spermatocyte are used to count the total number of cells in the tubule. The wall thickness of the tubule was measured at 3 locations in each picture (blue lines). (b) Cross-sectional area (CSA) of seminiferous tubules, the thickness of the cell layer of seminiferous tubules, and total cell number, Sertoli cells, and Leydig cells number per square millimeter in CON and db/db groups.

As shown in Figure 1(a), sperms were significantly reduced in the seminiferous tubules of db/db mice. db/db mice had a lower cross-sectional area (CSA) of seminiferous tubules and thickness of the cell layer than the control group (both *p* < 0.05). Compared with the control group, the total cell number (including germ cells and somatic cells) per square millimeter significantly decreased in db/db mice (4836.91 ± 261.80 vs. 5725.97 ± 199.26, *p*=0.013). Among somatic cells, both Sertoli cells (370.81 ± 18.32 vs. 589.82 ± 73.50, *p*=0.007) and Leydig cells (405.89 ± 38.41 vs. 272.18 ± 25.47, *p*=0.009) decreased in db/db mice (Figure 1(b)).

### 3.3. Cell Profiling in the Testis of db/db Mouse

**Figure 2:**
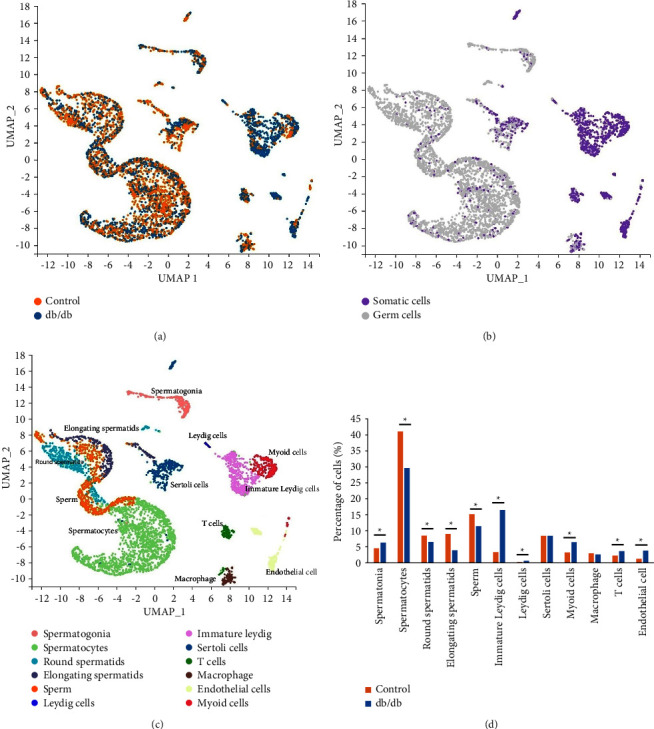
Cell clusters in db/db and C57BL/6J mice. Cell type analysis visualized with UMAP. Each dot represents a single cell. (a) Cells from db/db (blue) and C57BL/6J testis (orange). (b) Somatic cells (purple) and germ cells (grey). (c) Cell clusters were distinguished through marker genes and shown in different colors. (d) The proportion of each cell in db/db testis (blue) and C57BL/6J testis (orange), respectively. ^*∗*^*p* < 0.05.

In total, 18482 cells from the db/db (*n* = 9006) and control (*n* = 9476) groups were organized into 12 clusters, and the percentage of each cluster in the group was calculated. As shown in Figure 2, the db/db mouse had fewer germ cells and more somatic cells in the testis than controls. Compared with the control group, the percentage of spermatogonia was significantly higher in the db/db mouse (*p* < 0.001), while the proportions of spermatocytes, round and elongating spermatids, and sperms were all lower in the db/db mouse (*p* all <0.001). Among these cells, the number of elongating spermatids was the most significantly reduced cells in the testis of the db/db mouse (only 44.0% of the control group). Moreover, the proportions of immature and mature Leydig cells, myoid cells, endothelial cells, and T cells all significantly increased in the db/db mouse compared with the control (*p* all <0.001). The proportions of Sertoli cells and macrophages were similar between the two groups (both *p* > 0.05).

### 3.4. Differentially Expressed Genes in Germ Cells

**Figure 3:**
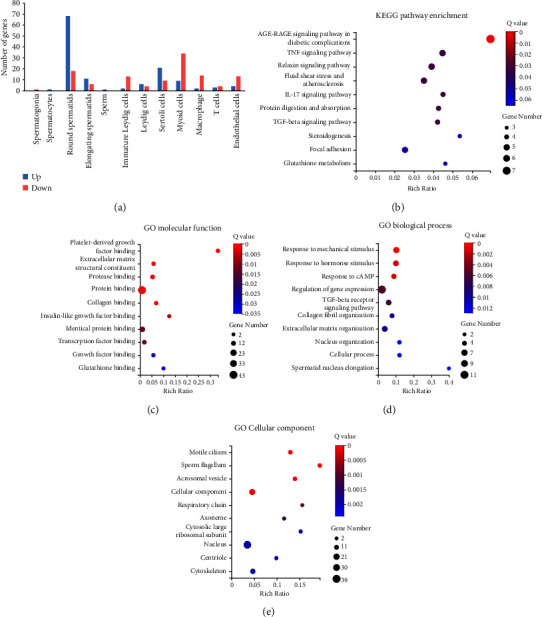
Differentially expressed genes in germ cells between db/db and C57BL/6J mice. (a) The numbers of differentially expressed genes (DEGs) between db/db and C57BL/6J mice in each cell cluster. Red represents upregulated DEGs, and blue represents downregulated DEGs in db/db testis compared with controls. (b–e) KEGG, GO molecular function, biological process, and cellular components enrichment analysis on the DEGs in spermatids.

We screened the differentially expressed genes by the Wilcox test with *p* < 0.05, log2 fold change (log2FC) > 0.25, and the percentage of cells with this gene (PCT) > 0.5. Finally, a total of 296 genes were screened out. Among the 12 clusters, the most differentially expressed genes were found in round spermatids (*n* = 86), and there was only one differentially expressed gene in spermatogonia (Prss50, log2fc = −0.31), spermatocyte (Jun, log2fc = 0.27), and sperm (Hbb-bs, log2fc = 0.88) separately (Figure 3(a)).

Among the germ cells, the differentially expressed genes were mainly in the clusters of spermatids. Therefore, we performed KEGG and GO enrichment analysis on the differentially expressed genes in spermatids (Figures 3(b)–3(d)). These genes were most correlated with the AGE-RAGE signaling pathway in diabetic complications in KEGG pathway analysis (Figure 3(b)). Their molecular functions were correlated with platelet-derived growth factor binding and insulin-like growth factor binding (Figure 3(c)). These genes were mainly involved in the biological process of response to mechanical and hormone stimuli (Figure 3(d)) and the cellular components of motile cilium and sperm flagellum (Figure 3(e)).

**Figure 4:**
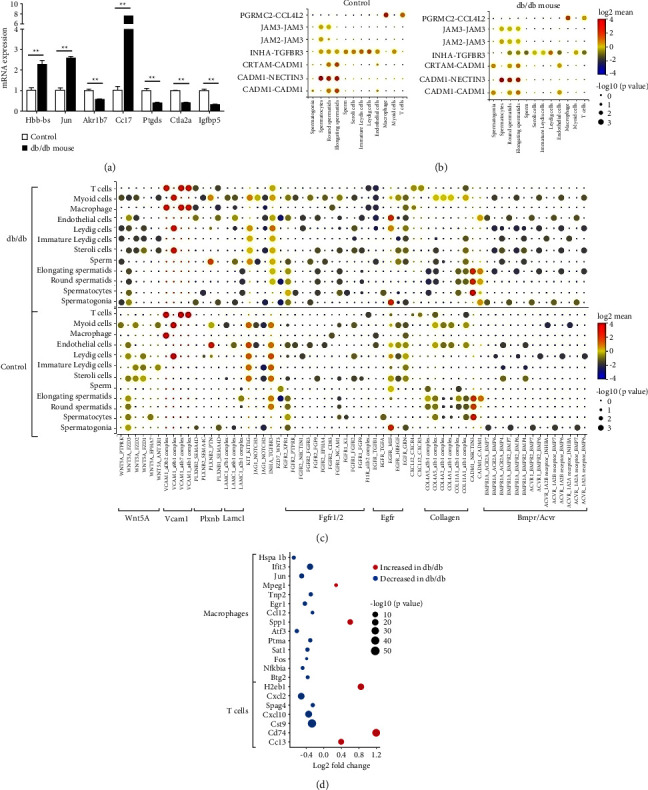
Differentially expressed genes between db/db and C57BL/6J mice. (a) Hbb-bs, Jun, Akr1b7, Igfbp5, Ctla2a, Ccl7, and Ptgds mRNA expression in the testicular tissue from db/db (*n* = 3) and C57BL/6J (*n* = 3) mice was tested using reverse transcription PCR; ^*∗∗*^*p* < 0.01. (b) Ligand-receptor interactions of Sertoli cells and other cells. The color (log 2 mean) of the circle represents the log of average expression level, and the size of the circle represents the *p* value; a larger point is more reliable. (c) Ligand-receptor interactions of Leydig cells and other cells. (d) The differentially expressed genes in Macrophages and T cells. The size of the circle represents the *p* value.

To confirm the results of single cell RNA-seq, we further performed reverse transcription-PCR with the testicular tissue from the remaining three mice in each group. As shown in Figure 4(a), Hbb-bs and Jun both significantly increased in db/db mice, and Akr1b7, the most decreased gene in round spermatids of db/db mice, also decreased in the PCR analysis (*p* all <0.01).

### 3.5. Differentially Expressed Genes in Leydig and Sertoli Cells

There were ten differentially expressed genes in Leydig cells (Table 2), which mainly secrete testosterone. Igfbp5 was the most significantly decreased gene in the db/db mouse, with the lowest *p* value of 8.13E-05. Ctla2a, a gene correlated with regulatory T cell differentiation and negative regulation of inflammatory response decreased most in the db/db mouse (log2fc = −1.73). The expression of Cd74, H2-Aa, and H2-Eb1, which were all associated with histocompatibility antigens, were elevated.

There were 31 differentially expressed genes between the db/db group and control in Sertoli cells. Among these genes, Ccl7 was the most significantly increased gene (*p*=7.34*E* − 34, log2fc = 0.38), and Ptgds was the most significantly decreased gene (*p*=1.28*E* − 28, log2fc = −0.49).

The mRNA expression of Igfbp5, Ctla2a, and Ptgds was also analyzed using reverse transcription-PCR, and these genes all significantly reduced in db/db mice (*p* all <0.01), and Ccl7 mRNA expression increased significantly (*p* < 0.001), which were consistent with the results of single cell RNA-seq (Figure 4(a)).

We analyzed the cell-cell communications between Sertoli/Leydig cells and other cells. As shown in Figures 4(b) and 4(c), the reduction of Inha expression in Sertoli/Leydig cells and Tgfbr3 in spermatids/sperm was observed in the db/db mouse. The expression of multiple ligand-receptor pairs, with the ligand of Wnt5a, Fgfr1/2, Col4a/11a, and Bmpr/Acvr in Leydig cells, decreased in the db/db mouse. The high expression of Vcam1-a4b1/7 complex between Leydig cells and macrophages was also identified in the db/db mouse, which was not observed in the control (Figure 4(c)).

### 3.6. The Differentially Expressed Genes in Immunology Cells

The expression of Cd74, Ccl3, and H2eb1 was higher in T cells of the db/db mouse than that of the control, and the expression of Cst9, Cxcl10, Cxcl2, and Spag4 decreased in the T cells of db/db mouse. In macrophages of the db/db mouse, Spp1 and Mpeg1 were significantly highly expressed, while the expression of Ifit3 and Hspa1b decreased the most (*p* all <0.05, Figure 4(d)).

## 4. Discussion

With the new technology of single-cell sequencing, the present study improved our understanding of the impairment in the testis of diabetic mice. In germ cells of db/db mice, the genes in spermatogonia, spermatocyte, and sperm are relatively conservative but change a lot in spermatids. According to the GO analysis, these abnormally expressed genes were mainly associated with motile cilium, sperm flagellum, acrosomal vesicles, centrioles, and other organelles, which all have important changes during sperm formation [25]. Therefore, aberrant expression of these genes may hinder the process of transformation of round spermatids into sperm.

The KEGG analysis in the present study found that the differentially expressed genes in spermatids between the db/db mice and normal controls were most correlated with the advanced glycation end product (AGE)-receptor for AGE (RAGE) signaling pathway in diabetic complications. AGEs are products of nonenzymatic glycation and oxidation of proteins, predominantly synthesized during chronic hyperglycemic conditions, and have been shown to accumulate in diabetic tissues [26]. RAGE, an immunoglobulin superfamily molecule, is the receptor of AGEs. The AGE-RAGE signaling pathway was also activated in renal [27], parotid gland [28], and retina [29] of db/db mice. Although AGE-RAGE signaling has been a well-studied cascade in diabetes, only one study showed that AGE-RAGE signaling pathway plays a role in sperm malformation and testis injury, and this study was performed in nondiabetic rats exposed to chlorpyrifos [30]. AGEs and RAGE were involved in inducing chronic immune imbalance in patients with diabetes. Such interaction attracts the immune cells into diffused glycated tissue and activates these cells to induce inflammatory damage [31]. However, both our study and the study of Sai et al. [30] proposed the role of the AGE-RAGE signaling pathway in testicular injury only by gene sequencing and KEGG analysis. The changes of this signaling pathway in testis and its effects on spermatogenesis in T2D remain to be further studied.

A previous study reported that db/db mice had lower testis weight, seminiferous tubule diameter, and seminiferous epithelium thickness than those of WT and db/+ mice at 12 and 24 weeks [32]. Our results are in keeping with this study and are also consistent with a study in rats exposed to a high-fat diet [33] but differ from the type 2 diabetic rats induced by feeding 10% fructose [10]. However, the histopathological change in the testis of patients with T2D has been rarely reported.

In addition to the germ cells, we also took a closer look at the somatic cells in the testis of db/db mice. Due to the significant reduction of germ cells, the proportion of most somatic cells increased in the single cell RNA-seq analysis, including Leydig cells. Although the proportion of Leydig cells in testicular cells increased, the number of Leydig cells remained significantly decreased in db/db mice in the histopathological analysis, which may cause the low level of testosterone. Igfbp5 encodes an insulin-like growth factor-binding protein and has been found highly expressed in Sertoli cells and Leydig cells of the testis [34]. Igfbp5 can activate or inhibit the IGF-PI3K-Akt signaling pathway in different tissues and cells [35]. However, the effects of Igfbp5 on the IGF signaling pathway in testis remain unclear. Previous studies have demonstrated that IGF-1 can regulate the differentiation of different testicular cells and has a synergistic effect with luteinizing hormone (LH) to regulate the number and function of Leydig cells [36]. Therefore, we speculate that the reduction of Igfbp5 expression may inhibit IGF-1 and leads to decreased Leydig cells and decreased testosterone synthesis. However, this hypothesis needs further study.

Ptgds encodes a protein called PGD2, which is expressed in Sertoli and germ cells, acts in both paracrine and autocrine manners, and contributes to the differentiation of germ cells [36, 37]. The influence of diabetes on the expression of PGD2 in the testis has not been reported before as we are aware of. A previous study found increased PGD2 content in brain damage in a rat model of T2D induced by STZ [38], which was the opposite of what we found in Sertoli cells of db/db mice. The decreased Ptgds expression may cause the inhibition of germ differentiation and lead to the disorder of spermiogenesis in the present study.

The present study found that several genes encoding histocompatibility antigens were highly expressed in Leydig cells and T cells, and several genes associated with autoimmune disease were also abnormally expressed in testis of db/db mice, such as Ctla2a, Ccl7, and Spp1. The abnormal expression of these genes indicated abnormal autoimmune responses [39, 40] and inflammation [41] in the testis of db/db mice. The proportion of T cells was also elevated in the testis of db/db mice in the present study. The autoimmune responses and inflammation may be caused by the injury of the blood testis barrier in diabetes [42] and can be aggravated by the accumulation of AGEs [31].

One limitation of this study is that we performed this study in db/db mice, which is a leptin receptor-deficient mouse model. Therefore, we cannot exclude the influence of the lack of leptin receptors in the testis. A previous study showed that in testis, leptin is mainly expressed in spermatocytes, and the leptin receptor is expressed in Leydig cells [43]. Leptin had an inhibition effect on testosterone production in mature Leydig cells, which leads to abnormal spermatogenesis [17, 43, 44]. Therefore, the disorder of steroidogenesis and spermatogenesis in db/db mice is more likely due to hyperglycemia and other metabolic problems but not the lack of leptin. Moreover, the leptin receptor expression is characteristic of mature Leydig cells and it is functional in adults but not prepubertal life [17], and the mutation of the leptin gene in db/db mice may not affect the testis composition during development. However, further study in diabetic patients and animal models without gene deficiency is necessary. A lack of protein analysis is also a limitation of the study since mRNA expression does not always reflect changes in protein levels and the phenotype of experimental animals. The effects of abnormal gene expression in this study on spermatogenesis and steroidogenesis in db/db mice need further investigation.

## 5. Conclusions

The present study indicates spermiogenesis and steroidogenesis defects in db/db mice. The mechanism of steroidogenesis impairment in the testis of db/db mice deserves further investigation.

## Figures and Tables

**Table 1:** The primers for real-time RT-PCR.

Genes	Forward	Reverse
Ccl7	CCATCAGAAGTGGGTCGAGG	ACCATTCCTTAGGCGTGACC
Ptgds	GCTCCTTCTGCCCAGTTTTC	CAGGAGGACCAAACCCATCC
Ctla2*α*	ACCGTGGACAACAAAATGATGG	TGCTTTTCTCTGCTCTCACCTG
Igfbp5	ACAGCTCTTTGCGCTCTCTT	GCGGGGTGATGGGTATACTG
Gapdh	TGAACGGGAAGCTCACTGG	TCCACCACCCTGTTGCTGTA
Hbb-bs	GCCCAGCACAATCACGA	TGCCTTTAACGATGGCCTGA
Jun	GAAGTGACGGACCGTTCTATGAC	GGAGGAACGAGGCGTTGAG
Akr1b7	CAGATTGAGAGCCACCCTTA	TGGGAATCTCCATTACTACG

**Table 2:** Differentially expressed genes in Leydig cells of db/db mouse.

Gene ID	Gene name	Official full name	Adjusted *p* value	log2fc	Pct
16011	Igfbp5	Insulin-like growth factor binding protein 5	8.13*E* − 05	−0.53907	0.908
12834	Col6a2	Collagen, type VI, alpha 2	4.81*E* − 04	−0.32879	0.892
13024	Ctla2a	Cytotoxic T lymphocyte-associated protein 2 alpha	0.007219	−1.72674	0.862
11816	Apoe	Apolipoprotein E	0.014743	−1.70878	0.554
16149	Cd74	CD74 antigen (invariant polypeptide of major histocompatibility complex, class II antigen associated)	1.64*E* − 09	0.677003	1
14960	H2-Aa	Histocompatibility 2, class II antigen A, alpha	1.64*E* − 09	0.609421	1
14969	H2-Eb1	Histocompatibility 2, class II antigen E beta	1.64*E* − 09	0.366559	1
52033	Pbk	PDZ binding kinase	0.005656	0.291496	1
100039095	Gm2044	lncRNA	0.005656	0.38096	1
8190	Mia	MIA SH3 domain containing	0.039967	0.531816	1

Log2fc: log2 fold change; Pct: the percentage of cells with this gene.

## Data Availability

The data used to support the findings of this study are available from the corresponding author upon reasonable request.
